# Catastrophic Health Expenditure and Its Determinants in Patients With Ischemic Heart Disease and Their Households: A Cross‐Sectional Study From Iran in 2024

**DOI:** 10.1002/hsr2.71082

**Published:** 2025-07-20

**Authors:** Mohammad Ali Laali Dehaghi, Sayed Saeed Kassaeian, Elahe Jandaghi, Farid Gharibi

**Affiliations:** ^1^ School of Medicine Semnan University of Medical Sciences Semnan Iran; ^2^ Department of Community Medicine, School of Medicine Semnan University of Medical Sciences Semnan Iran; ^3^ Department of Internal Medicine Semnan University of Medical Sciences Semnan Iran; ^4^ Social Determinants of Health Research Center Semnan University of Medical Sciences Semnan Iran; ^5^ Department of Health Services Management, School of Nursing Semnan University of Medical Sciences Semnan Iran

**Keywords:** catastrophic healthcare cost, financial accessibility, ischemic cardiovascular diseases, non‐communicable diseases

## Abstract

**Background:**

Ischemic heart diseases can lead to significant healthcare costs for patients due to their chronic and debilitating nature.

**Objective:**

The objective of this study was to examine the occurrence and causes of exorbitant healthcare expenses faced by individuals with ischemic heart disease and their households in Semnan in 2024.

**Methods:**

This cross‐sectional study included a cohort of 400 individuals diagnosed with angina and myocardial infarction who had received medical treatment for a minimum of 1 year. The data were collected using a researcher‐made questionnaire with content validity scores of 0.87 and 0.92 for the CVR (Content Validity Ratio) and CVI (Content Validity Index), respectively. The catastrophic healthcare costs were determined by evaluating the expenses incurred by patients and their households, and their annual income. This was done using a model that allocates 40% of nonfood household expenses to healthcare. Logistic regression analysis was used to assess the statistical associations between demographic and contextual characteristics and the occurrence of catastrophic healthcare costs.

**Results:**

The study reveals that patients and their households spend an average of 106,210,000 IRR (286.45 USD) annually on healthcare. Out of this amount, 80% is devoted to medical services, 16% to diagnostic services, and 4% to visits to different healthcare providers. Their mean yearly earnings amount to 1,061,430,000 IRR (2,862.72 USD), of which 297,580,000 IRR (802.58 USD) are allocated to nonfood expenditures. The mean proportion of direct medical expenses from nonfood household spending is 0.87 ( ± 1.50), and 46% of patients experience catastrophic healthcare costs. Significant statistical connections were observed between age, marital status, education, occupation, type of cardiac discomfort, duration of symptoms, initial diagnosis, and patient monitoring with catastrophic healthcare costs.

**Conclusions:**

Numerous demographic and contextual factors contribute to the incredibly high frequency of catastrophic healthcare costs among the patients and their households that were evaluated. Consequently, it is crucial to address this condition by conducting focused measures.

## 1 Introduction

Recent decades have seen a dramatic shift in the leading causes of morbidity and death from communicable and non‐communicable diseases (NCDs), as well as a fundamental change in the pattern of societies addressing diseases compared to the past [1]. Non‐communicable illnesses already account for more than 60% of all deaths globally [2], and their prevalence and impact are increasing [3]. In 2016, more than 287,000 people lost their lives due to NCDs [4], with 80% of those casualties happening in developing nations [2]. The World Health Organization (WHO) has called on nations to pledge to cut the number of early deaths caused by NCDs by 25% [5], citing the alarming rise in NCD‐related fatalities and disabilities as a significant public health concern [4].

Cardiovascular diseases (CVDs) are currently of great importance due to their extensive occurrence and adverse effects on both health and the economy. They contribute around 33% of all global fatalities [6], accounting for 12% of the worldwide disease burden [2]. The 2020 global report on CVD emphasizes a worrisome rise in their occurrence, death rates, and related impairments. The analysis reveals that between 1990 and 2019, there was a significant global increase in the number of individuals affected by CVDs, rising from 271 million to 523 million. Additionally, the number of deaths related to CVDs went from 12 million to 18.6 million, while the years lived with disability (YLDs) owing to CVDs rose from 17.7 million to 34.4 million [7].

It is worth noting that low‐ and middle‐income nations account for more than three‐quarters of CVD mortality [6], owing to an extraordinary increase in risk factors such as smoking, poor nutrition, and inadequate exercise [8]. In contrast to high‐income nations, where 80% of CVD‐related deaths occur in retirement, the majority of deaths in developing countries occur during productive ages [9, 10]. Developing nations have made CVD control a top health and social priority [11] but have had little success [2]. Furthermore, these countries' healthcare expenses are rising due to an aging population and dependence on expensive imported medical technologies [11].

Iran is among the nations with a high prevalence of CVDs as a result of shifting demographics, social structures, and epidemiological patterns. These illnesses are regarded as the primary cause of mortality in Iran [12, 13], accounting for about 50% of all fatalities and 80% of NCD‐related deaths [14, 15]. Ischemic cardiac disorders are a significant contributor to mortality and disability, accounting for 26% of deaths and 11% of Disability‐Adjusted Life Years (DALYs). Furthermore, the annual growth rates for mortality related to CVDs and the resulting DALYs are 8.6% and 3.4%, respectively [16]. The main factors contributing to this condition are the increasing age of the population, poor diet, lack of physical activity, and high rates of tobacco use [17]. A study in northern Iran examined the prevalence of risk factors of CVDs in people aged 40 to 60, and found that 96% of participants had at least one of the mentioned risk factors (including overweight or obesesity, high cholesterol levels, elevated triglycerides, high blood pressure, low HDL cholesterol, and high fasting blood sugar levels) [6].

It is worth noting that a significant percentage of cardiovascular illnesses, up to 80%, can be avoided by implementing appropriate therapies. This is supported by a decreasing pattern in the mortality rate of CVD in developed nations over the past few decades [18, 19], which can be linked to interventions in primary and secondary preventive measures [20]. Therefore, the success of a country's healthcare system in managing CVDs should focus on ensuring quality and accessible healthcare services (especially financially accessible) [21]. Imposing excessive healthcare costs can lead to inadequate or delayed access to essential healthcare services, ultimately undermining health outcomes and patient satisfaction [22, 23]. Given the importance of assessing financial access to healthcare services, particularly for individuals with chronic conditions such as CVD [24], and the lack of such assessments in Iran, the present study aimed to evaluate the extent and reasons for the financial burden faced by patients with ischemic heart diseases and their families.

## 2 Methods

### 2.1 Study Design and Participants

This cross‐sectional research was conducted in 2024, involving patients diagnosed with ischemic heart disease. The study's inclusion criteria were patients who had finished at least 1 year of their treatment and were experiencing myocardial infarction or angina. Simultaneous occurrence of other CVDs was the exit criterion for participants, as it could potentially confound the study results.

### 2.2 Sample Size

The sample size was calculated using the formula where the Z‐score for the specified confidence level is denoted by z, the anticipated variability in the responses is represented by s, and the margin of error is denoted by d:

$$n={\left(\frac{zs}{d}\right)}^{2}$$

Therefore, the sample size for this study is determined to be 384 cases at a 95% confidence level, with an anticipated variability of 0.5% and a margin of error of 0.05%. Considering the recommendation from some scientific sources to increase the sample size by 5%, 400 questionnaires were distributed. It is worth noting that calculating the sample size based on the Morgan table also yields a similar result.

$$n={\left(\frac{\left(1.96)(0.5\right)}{0.05}\right)}^{2}=\frac{0.9604}{0.0025}=384.16$$

### 2.3 Data Collection Tools

The data were collected using a researcher‐made questionnaire that underwent rigorous psychometric validation. Initially, cost components were identified through interviews with cardiovascular specialists, healthcare management professionals, and health economics experts. Patient care files were reviewed, and relevant studies were examined. These components were categorized into diagnostic, physician visits, and therapeutic domains. Subsequently, demographic and contextual variables potentially causing catastrophic costs were determined based on experts' opinions, and a preliminary questionnaire was created. Next, ten experts evaluated the questionnaire's content and face validity. All items in the initial questionnaire were assessed across the criteria of necessity, relevancy, clarity, and simplicity. The CVR and CVI were calculated based on the assessment results. CVR was determined using the obtained results for necessity, while CVI was calculated using results from the other three criteria, following the formula below:

$$\text{CVR}=\frac{\text{nE}-\frac{N}{2}}{\frac{N}{2}}$$

where nE is the number of experts choosing options with a positive load, and *N* is the sum of experts. In this study, due to the participation of 10 experts during the questionnaire development phase, a criterion of 0.62 acceptance score was used for assessment. The face validity of the instrument was also confirmed by obtaining expert feedback regarding the wording within the questionnaire. The final questionnaire consisted of 52 questions, with 17 items related to demographic/contextual independent variables, 31 questions concerning cost dimensions, and four regarding the patients and their household incomes. Based on expert evaluations, the final instrument's validity was confirmed with acceptance scores of 0.87 and 0.92 for CVR and CVI indices, respectively.

### 2.4 Data Collection Method and Data Analysis

The concept of catastrophic healthcare expenditures refers to patients' healthcare expenses surpassing their financial capacity to cover them [25]. When a patient and his household's nonfood spending exceeds healthcare costs by more than 40%, these expenses are classified as catastrophic healthcare costs [25]. Data on healthcare expenditures and patients' earnings from the previous year were gathered and scrutinized to investigate the occurrence of catastrophic healthcare expenses. The obtained healthcare expenditures are based on the exchange rates of the Central Bank of Iran, expressed in US dollars (a USD equivalent to 370,777 IRR on June 4, 2024). Interviews were done through both face‐to‐face interactions and telephone conversations, and each questionnaire was completed based on the patients' answers. Afterward, the ratio of medical expenses directly related to Nonfood household expenditures was calculated, and a minimum threshold of 40% was used as the criterion for determining when healthcare costs became catastrophic. The study utilized logistic regression analysis to examine the statistical association between demographic and contextual factors and catastrophic healthcare expenses. Both univariate (non‐adjusted) and multivariate (adjusted) logistic regression tests were employed. First, we assessed the statistical relationships between demographic and contextual variables and the occurrence of catastrophic healthcare costs. Variables with significance levels below 0.20 were studied more thoroughly using multivariate models and regression modeling. Ultimately, a reverse LR reduction technique was utilized to determine the ultimate relevant factors that impact the incidence of catastrophic healthcare costs.

### 2.5 Ethical Considerations

The researchers followed ethical norms, including respecting the patients' autonomy to choose whether or not to participate in the study, obtaining informed consent, guaranteeing patients' anonymity, and using the collected information solely for research reasons. Precautions were taken to ensure patient treatment processes were not disrupted throughout the data collection phase. Additionally, ethical approval (IR. SEMUMS. REC.1400.062) was acquired before conducting the research.

## 3 Results

The study was conducted in 2024 on patients with ischemic CVDs. Analysis of patient characteristics indicates that around 65% of the patients are aged between 50 and 69 years. The average age of the patients is 57.8 years, with a standard deviation of 12.65 years. The majority of them, precisely 69%, are male. Moreover, a significant proportion of patients are in a marital union (65%) or have experienced the loss of a spouse (24%). Approximately 78% of the individuals had not pursued higher education at a university, and more than 35% lacked basic literacy skills. The majority of patients have occupations as laborers (34%), homemakers (24%), or employees (19%). Most patients (79%) live in urban areas, and 80% are inhabitants of the province capital (Table 1).

**Table 1:** Demographic characteristics of patients.

Demographic characteristics	Grouping	Frequency	Percentage
Age	Less than 40 years	28	7
	40 to 49 years	47	11.75
	50 to 59 years	152	38
	60 to 69 years	109	27.25
	70 years and older	64	16
Gender	Female	122	30.5
	Man	278	69.5
Marital status	Single	32	8
	Married	261	65.25
	Separated	12	3
	Deceased wife	95	23.75
Educational level	Illiterate	140	35
	High school	33	8.25
	Diploma	140	35
	Associate and bachelor	74	18.5
	Masters	7	1.75
	PhD	6	1.5
Employment status	Employee	76	19
	Manual worker	136	34
	Self‐employed	13	3.25
	Retired	72	18
	Homemaker	96	24
	Unemployed	7	1.75
Urban/rural	Urban	316	79
	Rural	84	21
Residing in the provincial center	Yes	320	80
	No	80	20

The majority of patients (94%) have basic health insurance, predominantly from social security (83%), but less than a third (29%) have supplementary insurance. Most patients (84%) are solely affected by myocardial infarction, while 12% experience angina and 4% have both conditions concurrently. Only 63% of patients promptly sought medical attention and received a definitive diagnosis upon the onset of symptoms, yet the majority (86%) promptly began receiving necessary medical care following the initial diagnosis. For most patients (97%), the duration since the onset of illness has been less than 5 years, but only about two‐thirds (65%) are continuously monitored by their respective physicians. Cardiologists directly provide care to three‐fourths of the patients examined (75%), with general practitioners (21%) and internists (4%) sharing the responsibility. Additionally, most patients (86%) receive necessary services from private and public healthcare facilities concurrently (Table 2).

**Table 2:** Background characteristics of patients.

Demographic characteristics	Grouping	Frequency	Percentage
Basic insurance	Yes	376	94
	No	24	6
Type of basic insurance	Social security	311	82.7
	Health services	65	17.3
Supplemental insurance	Yes	118	29.5
	No	282	70.5
Type of heart disease	Angina	48	12
	Heart attack	336	84
	Both conditions	16	4
Time elapsed from the onset of symptoms to diagnosis	Immediately	254	63.5
	Less than one year	64	16
	1 to 4 years	73	18.25
	5 to 9 years	5	1.25
	Ten years or more	4	1
Time elapsed from diagnosis to start of care	Immediately	343	85.75
	Less than one year	53	13.25
	1 to 4 years	4	1
Time elapsed since the onset of the disease	Less than five years	388	97
	5 to 10 years	12	3
Monitoring status	Continuous	260	65
	Intermittent	86	21.5
	In case of serious problems	54	13.5
Attending physician	General practitioner	84	21
	Internal medicine specialist	17	4.25
	Cardiologist	299	74.75
Place of care	Public centers	24	6
	Private centers	32	8
	Combination of public and private centers	344	86

The analysis of imposed costs on patients shows that, on average, they spend 16,470,000 IRR (44.42 USD) annually on diagnostic expenses. Angiography, echocardiography, and laboratory services account for 48%, 30%, and 20% of these expenses. Calculations of the costs imposed on patients for visits to various healthcare providers indicate an average annual expenditure of 4,430,000 IRR (11.95 USD) per patient on visits. Most of this (81%) is spent on consultations with cardiovascular specialists. Furthermore, patients spend an average of 85,290,000 IRR (230.03 USD) annually on therapeutic care, with medication and hospitalization accounting for 57% and 35% of these expenses, respectively. Overall, these patients annually spend 106,210,000 IRR (286.45 USD) on healthcare services, with 80% allocated to therapeutic services, 16% to diagnostic services, and 4% to visits to various healthcare providers (Table 3).

**Table 3:** Direct medical costs imposed on patients.

Types of care costs		Minimum^a^	Maximum^b^	Mean	St. dev.
		IRR (USD)	IRR (USD)	IRR (USD)	± IRR (USD)
***Diagnostic services***	Laboratory	0	60,000,000 (161.82)	3,381,350 (9.12)	5,399,000 (14.56)
	Angiography	0	600,000,000 (1,618.22)	7,934,000 (21.40)	44,858,910 (120.98)
	CT Scan	0	7,000,000 (18.88)	42,500 (0.11)	505,680 (1.36)
	Echo	0	40,000,000 (107.88)	5,007,000 (13.50)	6,330,570 (17.07)
	Radiography	0	6,000,000 (16.18)	52,500 (0.14)	479,200 (1.29)
	ECG	0	8,500,000 (22.92)	61,250 (0.16)	611,860 (1.65)
	***Total***	***0***	***609,500,000 (1,643.84)***	***16,478,600 (44.44)***	***45,951,150 (123.93)***
***Consultation fees***	General practitioner	0	16,000,000 (43.15)	417,750 (1.12)	1,291,610 (3.48)
	Internal medicine specialist	0	6,900,000 (18.61)	129,500 (0.35)	686,730 (1.85)
	Cardiologist	0	48,000,000 (129.46)	3,590,850 (9.68)	4,675,530 (12.61)
	Heart surgeon	0	7,000,000 (18.88)	299,000 (0.81)	883,110 (2.38)
	***Total***	***0***	***48,000,000 (129.46)***	***4,437,100 (11.97)***	***5,061,290 (13.65)***
***Treatment services***	Surgery	0	140,000,000 (377.58)	2,665,000 (7.19)	14,077,770 (37.97)
	Hospitalization	0	507,000,000 (1,367.40)	30,315,000 (81.76)	53,626,460 (144.63)
	Medication	0	700,000,000 (1,887.93)	48,857,500 (131.77)	62,082,410 (167.44)
	Supplementary drugs	0	300,000,000 (809.11)	3,461,000 (9.33)	22,298,120 (60.13)
	***Total***	***0***	***700,000,000 (1,887.93)***	***85,298,500 (230.05)***	***88,697,530 (239.22)***
**Total costs**		**1,300,000 (3.50)**	**755,000,000 (2,036.26)**	**106,214,200 (286.46)**	**103,128,040 (278.14)**

a The least amount of money paid to receive needed health care from patients and their households.

b The largest amount of money paid to receive needed health care from patients and their households.

The examination of patients and their household income reveals that their average annual income is 1,061,430,000 IRR (2,862.72 USD), with 297,580,000 IRR (802.58 USD) spent on Nonfood expenditures annually. The average healthcare costs constitute 87% ( ± 1.50) of these Nonfood expenditures. Approximately 46% of these households face catastrophic healthcare costs (Table 4).

**Table 4:** Annual income status of patients and their families.

Household income and expenses	Minimum	Maximum	Mean	St. dev.
	IRR (USD)	IRR (USD)	IRR (USD)	IRR (USD)
Income	60,000,000 (161.82)	9,600,000,000 (25,891.57)	1,061,430,000 (2,862.71)	872,736,380 (2,353.80)
Food expenses	20,000,000 (53.94)	2,400,000,000 (6,472.89)	681,700,000 (1,838.57)	334,686,690 (902.66)
Nonfood expenses	32,000,000 (86.30)	2,400,000,000 (6,472.89)	297,580,000 (802.58)	295,279,000 (796.38)
Savings	0	1,200,000,000 (3,236.45)	42,550,000 (114.76)	162,177,380 (437.40)

In single‐variable regression analysis, a significant statistical relationship was found among the variables of age, marital status, education, occupation, type of cardiac discomfort, duration from symptom onset to initial diagnosis, and patient monitoring with the incidence of catastrophic healthcare expenses (*p* < 0.05). More precisely, the probability of experiencing extremely high healthcare costs rises by 110% for every distinct age group change, 84% for changes in marital status, 52% for variations in education level, 38% for different occupations, 40% for various types of cardiac discomfort, 187% for changes in the duration from symptom onset to initial diagnosis, and by 69% for variations in patient monitoring. In the multiple‐variable regression analysis, only the education level variable showed significance, indicating that the chance of catastrophic healthcare expenses increases by 43% for each higher education category among patients. In the final reduced model, age and education level were identified as the main influential variables affecting the incidence of catastrophic healthcare expenses. Specifically, each age category increase leads to a 56% higher chance of catastrophic healthcare expenses (facilitative effect due to OR being greater than 1), and each education level category increase results in a 41% lower incidence in catastrophic healthcare expenditures (protective effect due to OR being less than 1) (Table 5).

**Table 5:** Statistical significance of demographic and contextual variables with catastrophic healthcare costs.

Variables	Univariate model		Multiple model		Reduced model			
	Non‐adjusted OR	*p* value	Adjusted OR	*p* value	Adjusted OR	95% CI for OR		*p* value
						Lower	Upper	
Age	2.106	< 0.001	1.296	0.266	1.560	1.102	2.210	0.012
Gender	0.626	0.129	1.033	0.954				
Marital status	1.841	< 0.001	1.292	0.271				
Education	0.481	< 0.001	0.575	0.004	0.590	0.435	0.799	0.001
Occupation	1.385	0.001	1.118	0.578				
Residence	1.571	0.204						
Urban/rural	1.561	0.202						
Basic insurance	1.696	0.381						
Type of basic insurance	1.993	0.081	1.116	0.810				
Supplementary insurance	1.500	0.199	0.550	0.162				
Type of heart disease	1.400	0.038	1.745	0.242				
Symptoms to diagnosis	2.878	0.011	0.907	0.673				
Diagnosis to care	1.901	0.090	1.207	0.701				
Time elapsed	2.409	0.317						
Under observation	1.693	0.010	0.941	0.821				
Responsible physician	0.895	0.523						
Place of care	1.107	0.708						

*Note:* Grey cells refer to variables that were excluded from further analyses due to a significance level of more than 0.2.

## 4 Discussion

The present study investigated the incidence and reasons influencing catastrophic healthcare expenses among patients with ischemic heart disease and their households in Semnan City. Nearly two‐thirds of the patients (65%) fall within the age range of 50 to 69 years. However, patients under 40 and those aged 40 to 49 constitute 7% and 12% of the patient population, respectively, indicating a decreasing trend in the age of onset for these diseases. The results of various studies suggest that the incidence of CVDs and even their severity are directly related to increasing age [17, 26]. As the population ages, DALYs related to CVD will more than double from 2005 to 2025 [2]. Moreover, over two‐thirds of the patients are male (69%), which could be attributed to a higher prevalence of cardiovascular risk factors such as smoking and occupational stress among men compared to women [17, 26]. This might also reflect men's lesser attention to their health, resulting in delayed medical attention to symptoms and less adherence to medical care advice [27].

Another notable point is that 35% of the patients are illiterate, and 78% lack university education, which could make educating them about self‐care practices more challenging. Having basic and academic literacy can lead to improved health literacy, which can enhance the level of self‐care in patients with CVDs and improve their health [28]. Most patients (94%) have basic health insurance, but less than one‐third (29%) have supplementary insurance. Considering the universal health insurance (UHC) law, 6% of patients without basic insurance will likely face difficulties. Additionally, the absence of supplementary health insurance in 71% of patients could lead to reduced financial access to care for them. Benefiting from appropriate health insurance designed based on the principles of universal health coverage (full coverage of the population, their needed care, and their medical costs) can remove financial barriers to accessing health services and subsequently lead to increased health levels and patient satisfaction [29].

Only 63% of patients sought immediate follow‐up and definitive diagnosis immediately after symptoms appeared. However, most (86%) promptly sought appropriate care after their initial diagnosis. The lack of initial follow‐up for disease symptoms, leading to diagnosis, is evident in 37% of the patients. Additionally, 14% of them do not receive necessary care, highlighting significant deficiencies likely stemming from financial constraints preventing patients from accessing care promptly and underestimating the seriousness of CVDs and the importance of timely follow‐up. Only about two‐thirds (65%) of them are regularly monitored by their respective physicians, and 21% receive necessary care from general practitioners. These percentages are not satisfactory, given the sensitivity and complexity of ischemic heart diseases. It is evident that providing effective health education, having a good level of health literacy and self‐care, as well as effective health insurance, can minimize delays in diagnosing disease and receiving timely medical care [28, 29]. Most patients (86%) receive required services from private and government healthcare facilities simultaneously. This may indicate insufficient (and possibly lower quality) care provided in the public sector [6].

Each year, patients pay an average of 106,210,000 IRR (286.45 USD) as direct medical expenses. Of this amount, 80% is allocated to healthcare services, especially medication and hospitalization; 16% goes to diagnostic services, particularly angiography, echocardiography, and laboratory tests; and 4% is spent on visits to various healthcare providers, notably cardiologists and specialists in vascular diseases. The average annual income of these patients is 1,061,430,000 IRR (2,862.72 USD), with 297,580,000 IRR (802.58 USD) spent on nonfood expenses. On average, medical expenses constitute 0.87 ( ± 1.50%) of their nonfood household expenses, indicating a significant financial burden. Half of the patients face catastrophic healthcare expenses. The regression analysis found a statistically significant relationship between variables such as age, marital status, education, occupation, type of cardiac discomfort, duration from symptom onset to initial diagnosis, patient monitoring, and the incidence of catastrophic healthcare expenses. However, age and education level were identified as the most influential variables.

The study by Mohanan in 2019 in India reported that 54% of patients with acute myocardial infarction face catastrophic health expenditures. The study found statistically significant associations between having health insurance and household income levels with the incidence of catastrophic health expenditures. Individuals without health insurance were 24% more likely to face catastrophic health expenditures and were three times more likely to encounter financial difficulties [30]. A study by Jan in 2012 across Asian countries, including China, India, Malaysia, South Korea, Singapore, Thailand, and Vietnam, showed that the incidence of catastrophic health expenditures among hospitalized patients with acute coronary syndrome was 52% for those with health insurance and 66% for those without health insurance [22]. The occurrence rates varied widely, ranging from 56% to 80% for insured and uninsured patients in China to 0% in Malaysia [22]. Daivadanam's study in 2011 in India estimated that 84% of patients with acute coronary syndrome incur catastrophic health expenditures [31]. It identified household financial status, employment status, and health insurance as influential factors affecting the occurrence of catastrophic health expenditures [31].

A study by Si in 2013 in China demonstrated that 24% of patients with hypertension face catastrophic health expenditures. If these individuals also suffer from other NCDs like diabetes, their incidence of facing catastrophic health expenditures rises to 34% [32]. A study by Zhang (2020) in China estimated catastrophic health expenditures and poverty to be 13.6% and 10.8%, respectively [33]. Additionally, the study estimated the occurrence of catastrophic health expenditures in patients with one cardiovascular condition at 25.3% and those with two or more conditions at 47.3%. At the same time, it was reported that 6.1% of patients had no conditions [33]. This study identified background variables such as income level, household size, and education level as significant factors affecting the incidence of catastrophic health expenditures [33]. Imamgholipour's study in 2015 in Ahvaz, Iran, reported that 55% of patients with CVDs face catastrophic health expenditures. The study identified income level, household size, and employment status as primary factors influencing the incidence of catastrophic health expenditures [34]. A review of published literature indicates that CVDs, especially ischemic heart diseases like angina and heart attacks, impose significant and often catastrophic financial burdens on patients and their households. These burdens can vary depending on the level of development of healthcare and the social and economic systems governing it. Income level and health insurance are highlighted as primary determinants of the incidence of catastrophic health expenditures among households with members suffering from CVDs.

A multifaceted approach is required to address CVDs effectively. The following executive recommendations provide a comprehensive strategy to prevent and manage ischemic CVDs across various stages of life:

1. Universal Education Programs
  Implement effective education programs targeting ischemic CVD prevention from early childhood through young adulthood (the first three decades of life).
2. Screening Programs

− Initiate regular screening programs from the fourth decade of life.
− Focus on identifying high‐risk patients, especially in their sixth and seventh decades.
− Prioritize screenings for men, particularly those with identified risk factors.

3. Accessibility and Continuity of Education

− Ensure men have continuous access to relevant prevention and self‐care education.
− Provide comprehensive and ongoing education on CVD symptoms and the importance of timely medical follow‐ups.

4. Insurance Coverage

− Ensure comprehensive primary insurance coverage and expand supplementary insurance for patients.
− Evaluate and improve insurance effectiveness based on the three dimensions of universal health coverage: covering all individuals, services, and costs.

5. Community Education

− Educate the broader community, with a focus on high‐risk groups, about CVD symptoms and the necessity of timely medical follow‐ups for proper care.

6. Patient Education

− Inform patients about the importance of continuous supervision by qualified physicians.

7. Healthcare Services Expansion

− Expand necessary care services for CVD patients quantitatively and qualitatively within the public healthcare sector.

8. Support for Knowledge‐Based Companies

− Encourage and support local knowledge‐based companies in producing essential drugs and diagnostic equipment, ensuring appropriate quality and quantity.

9. Addressing Health Sanctions

− Work towards lifting unjust health sanctions, especially those affecting the pharmaceutical industry.

10. Referral System Reform

− Reform the referral system to include cardiovascular specialists, which would help reduce the financial burden of catastrophic health expenditures on patients.

In addition to the above implementation suggestions to reduce the burden of disease and its costs on patients and the health system, the following research suggestions can also be presented:

− Conduct similar studies in other regions of the country to determine the occurrence of backbreaking health costs in patients with ischemic CVDs and how they are distributed.
− Repeat the present study periodically at specific time intervals to identify the disease trend, and use statistical modeling to make accurate predictions in this regard.
− Conduct similar studies on other diseases with a high probability of occurrence of backbreaking health costs, such as chronic debilitating and costly diseases.
− Implement intervention studies based on the suggestions in this study to enhance the situation and share the resulting experiences.

The study's disadvantage is that it relies on patients' memory to recall expenses incurred during the previous year, which increases the possibility of recall bias. This constraint derives from the lack of a complete health information system for tracking patient treatment and expenses. However, this disadvantage was overcome by evaluating patients' medical records and documentation throughout the data collection phase.

## 5 Conclusion

The results obtained from this study indicate that the incidence of catastrophic health expenditures among patients with ischemic heart disease is not satisfactory, suggesting the need for intervention and improvement measures to address this issue. It is clear that in the design and implementation of supportive interventions, focusing on preventive actions and paying particular attention to patients facing higher exposure can enhance the effectiveness and efficiency of implemented interventions in a targeted manner.

## Author Contributions

**Mohammad Ali Laali Dehaghi:** conceptualization, methodology, formal analysis, writing – original draft, investigation. **Sayed Saeed Kassaeian:** conceptualization, data curation, methodology, investigation, validation, writing – original draft. **Elahe Jandaghi:** conceptualization, formal analysis, writing – review and editing, software, methodology. **Farid Gharibi:** conceptualization, investigation, supervision, software, validation, methodology, writing – review and editing, project administration.

## Conflicts of Interest

The authors declare no conflicts of interest.

## 1 Transparency Statement

Farid Gharibi affirms that this manuscript is an honest, accurate, and transparent account of the study being reported, that no important aspects of the study have been omitted, and that any discrepancies from the study as planned (and, if relevant, registered) have been explained.

## Data Availability

The data that support the findings of this study are available on request from the corresponding author. The data are not publicly available due to privacy or ethical restrictions.

## References

[1] T. Kazemi , M. Hajihosseini , M. Moossavi , M. Hemmati , and M. Ziaee , “Cardiovascular Risk Factors and Atherogenic Indices in an Iranian Population: Birjand East of Iran,” Clinical Medicine Insights: Cardiology 12 (2018): 1–6. 10.1177/1179546818759286 PMC5824902 29497341

[2] M. Sadeghi , A. A. Haghdoost , A. Bahrampour , and M. Dehghani , “Modeling the Burden of Cardiovascular Diseases in Iran From 2005 to 2025: The Impact of Demographic Changes,” Iranian Journal of Public Health 46, no. 4 (2017): 506–516. 28540267 PMC5439040

[3] C. D. Mathers and D. Loncar , “Projection of Global Mortality and Burden of Disease From 2002 to 2030,” PLoS Medicine 3, no. 11 (2006): e442. 17132052 10.1371/journal.pmed.0030442 PMC1664601

[4] N. Peykari , H. Hashemi , G. Asghari , et al., “Scientometric Study on Non‐Communicable Diseases in Iran: A Review Article,” Iranian Journal of Public Health 47, no. 7 (2018): 936–943. 30181990 PMC6119576

[5] A. Alwan , “The World Health Assembly Responds to the Global Challenge of Noncom‐Municable Diseases,” Eastern Mediterranean Health Journal 19, no. 6 (2013): 511–512. 24975178

[6] S. Mouodi , S. R. Hosseini , R. Graham Cumming , A. Bijani , H. Esmaeili , and R. Ghadimi , “Physiological Risk Factors for Cardiovascular Disease in Middle‐Aged (40–60 Year) Adults and Their Association With Dietary Intake, Northern Iran,” Caspian Journal of Internal Medicine 10, no. 1 (2019): 55–64. 30858942 10.22088/cjim.10.1.55 PMC6386329

[7] G. A. Roth , G. A. Mensah , C. O. Johnson , et al., “Global Burden of Cardiovascular Diseases and Risk Factors, 1990–2019,” Journal of the American College of Cardiology 76, no. 25 (2020): 2982–3021. 33309175 10.1016/j.jacc.2020.11.010 PMC7755038

[8] M. F. Kilkenny , L. Dunstan , D. Busingye , et al., “Knowledge of Risk Factors for Diabetes or Cardiovascular Disease (CVD) is Poor Among Individuals With Risk Factors for CVD,” PLoS One 12 (2017): e0172941. 28245267 10.1371/journal.pone.0172941 PMC5330511

[9] D. O. Abegunde , C. D. Mathers , T. Adam , M. Ortegon , and K. Strong , “The Burden and Costs of Chronic Diseases in Low‐Income and Middle‐Income Countries,” Lancet 370 (2007): 1929–1938. 18063029 10.1016/S0140-6736(07)61696-1

[10] B. J. Gersh , K. Sliwa , B. M. Mayosi , and S. Yusuf , “Novel Therapeutic Concepts: The Epidemic of Cardiovascular Disease in the Developing World: Global Implications,” European Heart Journal 31 (2010): 642–648. 20176800 10.1093/eurheartj/ehq030

[11] H. L. Walls , A. Peeters , C. M. Reid , D. Liew , and J. J. McNeil , “Predicting the Effectiveness of Prevention: A Role for Epidemiological Modeling,” Journal of Primary Prevention 29 (2008): 295–305. 18581234 10.1007/s10935-008-0143-y

[12] M. Naghavi , F. Abolhassani , F. Pourmalek , et al., “The Burden of Disease and Injury in Iran 2003,” Population Health Metrics 7, no. 9 (2009): 9. 19527516 10.1186/1478-7954-7-9 PMC2711041

[13] M. T. Sheykhi , “General Review of Sociologi‐Cal Challenge and Prospects of Population in Iran a Sociological Study of Quality of Life,” Journal of Social Sciences 12 (2006): 21–32.

[14] Z. N. Hatmi , S. Tahvildari , A. Gafarzadeh Motlagh , and A. Sabouri Kashani , “Prevalence of Co‐Ronary Artery Disease Risk Factors in Iran: A Population Based Survey,” BMC Cardiovascular Disorders 7, no. 3 (2007): 1–10. 17971195 10.1186/1471-2261-7-32 PMC2200651

[15] N. Fahimfar , D. Khalili , S. G. Sepanlou , et al., “Cardiovascular Mortality in a Western Asian Country: Results From the Iran Cohort Consortium,” BMJ Open 8 (2018): e020303. 10.1136/bmjopen-2017-020303 PMC6042599 29980541

[16] GBD . Available at: vizhub.healthdata.org/gbd-compare/.

[17] F. Hosseini‐Esfahani , A. Mousavi Nasl Khameneh , P. Mirmiran , A. Ghanbarian , and F. Azizi , “Trends in Risk Factors for Cardiovascular Disease Among Iranian Adolescents: The Tehran Lipid and Glucose Study, 1999–2008,” Journal of Epidemiology 21 (2011): 319–328. 21804294 10.2188/jea.JE20100162 PMC3899430

[18] E. S. Ford and S. Capewell , “Coronary Heart Dis‐Ease Mortality Among Young Adults in the US From 1980 Through 2002: Concealed Levelling of Mortality Rates,” Journal of the American College of Cardiology 50 (2007): 2128–2132. 18036449 10.1016/j.jacc.2007.05.056

[19] R. Joshi , S. Jan , Y. Wu , and S. MacMahon , “Global Inequalities in Access to Cardiovascular Health Care,” Journal of the American College of Cardiology 52 (2008): 1817–1825. 19038678 10.1016/j.jacc.2008.08.049

[20] T. A. Gaziano , “Reducing the Growing Burden of Cardiovascular Disease in the Developing World,” Health Affairs 26 (2007): 13–24. 17211010 10.1377/hlthaff.26.1.13 PMC2365905

[21] A. Imani , F. Gharibi , A. Khezri , N. Joudyian , and K. Dalal , “Economic Costs Incurred by the Patients With Multiple Sclerosis at Different Levels of the Disease: A Cross‐Sectional Study in Northwest Iran,” BMC Neurology 20, no. 205 (2020): 205. 32446303 10.1186/s12883-020-01790-5 PMC7245021

[22] S. Jan , S. W.‐L. Lee , J. P. Sawhney , et al., “Catastrophic Health Expenditure on Acute Coronary Events in Asia: A Prospective Study,” Bulletin of the World Health Organization 94 (2016): 193–200. 26966330 10.2471/BLT.15.158303 PMC4773930

[23] Z. Jing , J. Chu , Z. Imam Syeda , et al., “Catastrophic Health Expenditure Among Type 2 Diabetes Mellitus Patients: A Province‐Wide Study in Shandong, China,” Journal of Diabetes Investigation 10, no. 2 (2019): 283–289. 30044060 10.1111/jdi.12901 PMC6400173

[24] A. Haakenstad , M. Coates , A. Marx , G. Bukhman , and S. Verguet , “Disaggregating Catastrophic Health Expenditure by Disease Area: Cross‐Country Estimates Based on the World Health Surveys,” BMC Medicine 17, no. 36 (2019): 36. 30755209 10.1186/s12916-019-1266-0 PMC6372998

[25] L. C. Chin , B. Li , and G. Murray , “CJL. Limitations of Methods for Measuring Out‐of‐Pocket and Catastrophic Private Health Expenditures,” Bulletin of the World Health Organization 87 (2009): 238–244. 19377721 10.2471/BLT.08.054379 PMC2654642

[26] M. F. Kilkenny , L. Dunstan , D. Busingye , et al., “Knowledge of Risk Factors for Diabetes or Cardiovascular Disease (CVD) Is Poor Among Individuals With Risk Factors for CVD,” PLoS One 12, no. 2 (2017): e0172941. 28245267 10.1371/journal.pone.0172941 PMC5330511

[27] E. M. Hernandez , R. Margolis , and R. A. Hummer , “Educational and Gender Differences in Health Behavior Changes After a Gateway Diagnosis,” Journal of Aging and Health 30, no. 3 (2018): 342–364. 27940641 10.1177/0898264316678756 PMC5777891

[28] M. I. Cajita , T. R. Cajita , and H. R. Han , “Health Literacy and Heart Failure: A Systematic Review,” Journal of Cardiovascular Nursing 31, no. 2 (2016): 121–130. 25569150 10.1097/JCN.0000000000000229 PMC4577469

[29] D. A. Watkins and R. A. Nugent , “Setting Priorities to Address Cardiovascular Diseases Through Universal Health Coverage in Low‐ and Middle‐Income Countries,” Heart Asia 9, no. 1 (2017): 54–58. 28321266 10.1136/heartasia-2015-010690 PMC5337683

[30] P. P. Mohanan , M. D. Huffman , A. S. Baldridge , et al., “Microeconomic Costs, Insurance, and Catastrophic Health Spending Among Patients With Acute Myocardial Infarction in India Substudy of a Randomized Clinical Trial,” JAMA Network Open 2, no. 5 (2019): e193831. 31099866 10.1001/jamanetworkopen.2019.3831 PMC6537817

[31] M. Daivadanam , K. R. Thankappan , P. S. Sarma , and S. Harikrishnan , “Catastrophic Health Expenditure & Coping Strategies Associated With Acute Coronary Syndrome in Kerala, India,” Indian Journal of Medical Research 136 (2012): 585–592. 23168698 PMC3516025

[32] Y. Si , Z. Zhou , M. Su , M. Ma , Y. Xu , and J. Heitner , “Catastrophic Healthcare Expenditure and Its Inequality for Households With Hypertension: Evidence From the Rural Areas of Shaanxi Province in China,” International Journal for Equity in Health 16, no. 27 (2017): 27. 28666448 10.1186/s12939-016-0506-6 PMC5493855

[33] X. Zhang , Q. Xu , X. Guo , et al., “Catastrophic Health Expenditure: A Comparative Study Between Hypertensive Patients With and Without Complication in Rural Shandong, China,” BMC Public Health 20, no. 5 (2020): 545. 32321485 10.1186/s12889-020-08662-0 PMC7178564

[34] S. Emamgholipour , A. Akbari Sari , S. Geravandi , and H. Mazrae , “Estimation of Out‐of‐Pocket and Catastrophic Health Expenditure Among Patients With Cardiovascular Disease in Khuzestan,” Payavard 11, no. 3 (2017): 297–307.

